# Cholesteric Liquid Crystal Elastomer-Based Single-Ion Conductor for Advanced Quasi-Solid Electrolyte Membranes

**DOI:** 10.3390/molecules31162879

**Published:** 2026-08-18

**Authors:** Tangqi Hu, Junxian Fu, Yonggang Yang, Yi Li

**Affiliations:** Jiangsu Key Laboratory of Advanced Functional Polymer Materials, Department of Polymer Science and Engineering, College of Chemistry, Chemical Engineering and Materials Science, Soochow University, Suzhou 215123, China

**Keywords:** lithium-ion batteries, quasi-solid electrolytes, cholesteric liquid crystal elastomers, single-ion conductivity, directional transport

## Abstract

Self-assembled liquid crystal polymer networks build directional transport channels via molecular alignment, enabling efficient and ordered lithium-ion migration, while single-ion conducting polymer electrolytes improve lithium-ion transference number by covalently anchoring anions, which alleviates concentration polarization and effectively suppresses lithium dendrite growth. Herein, a series of substrate-free Li salt-grafted cholesteric liquid crystal elastomers (CLCE) with tunable helical pitches and spiral orientation were fabricated, and quasi-solid electrolyte membranes were obtained through plasticization. It was found that samples with the smallest helical pitch delivered the highest lithium-ion transference number irrespective of the spiral orientation. The optimal CLCE electrolyte membrane achieved a lithium-ion transference number of 0.94, exhibiting characteristic single-ion conductivity. Moreover, after adding small amount of extra free Li salt, the prepared electrolyte membrane delivered a room-temperature ionic conductivity of 2.6 × 10^−4^ S·cm^−1^, an electrochemical stability window of 5.2 V and stable cycling performance, providing new insights into the design of high-performance safe quasi-solid lithium-ion batteries.

## 1. Introduction

The global transition toward low-carbon energy systems has rendered lithium-ion batteries (LIBs) strong candidates for next-generation high-energy-density energy storage systems, owing to the ultrahigh theoretical specific capacity and the lowest electrochemical potential of lithium metal. However, the inherent risks of leakage, flammability and thermal runaway in conventional liquid electrolytes, coupled with intractable dendrite growth during repeated lithium plating/stripping processes, severely restrict the safety and cycling lifespan of LIBs [1,2,3]. Quasi-solid electrolytes (QSEs), which combine the high ionic conductivity of liquid systems and the mechanical barrier capability of solid-state counterparts, are regarded as a critical approach to improve the safety and stability of LIBs [4]. In QSE systems, single-ion conductors covalently anchor anions onto the polymer backbone, enabling a lithium-ion transference number (***t***_Li_^+^) approaching unity. This design effectively eliminates concentration polarization, suppresses lithium dendrite growth and markedly enhances the cycling stability, thus attracting extensive attention in recent years [5,6]. For instance, Van Vliet et al. synthesized borate-backbone single-ion polymer electrolytes via the step-growth polymerization of borane precursors and dilithium-substituted benzene. The anionic skeletons are firmly immobilized by covalent bonds, which fundamentally reduces the concentration polarization caused by anion migration. Meanwhile, they compared the effects of three bridging units (oxygen, sulfur and methylene) on ion transport performance, refining the molecular design strategy for boron-based single-ion conductors [7]. Yu et al. fabricated fluorinated grafted single-ion copolymers, achieving a high ***t***_Li_^+^ of 0.94, significantly improving electrode–electrolyte interfacial stability, and achieving excellent cycling performance in high-loading full cells [8]. These studies demonstrate the great potential of single-ion conductor design strategies in boosting the electrolyte performance of lithium metal batteries.

Although single-ion conductors can substantially mitigate concentration polarization and lithium dendrite issues, the disordered ion transport pathways in conventional random polymer electrolytes still impose a notable bottleneck on ion conduction efficiency. Among various strategies for constructing ordered ion transport channels, cholesteric liquid crystals (CLCs) stand out for their unique periodic helical superstructure. CLC molecules rotate continuously along the helical axis, forming a chiral nematic arrangement with a characteristic pitch (***P***). This one-dimensional helical topology can build continuous and anisotropic ion migration pathways at the microscopic scale [9,10,11,12]. In particular, the helical arrangement of cholesteric phases not only provides a physical framework for oriented conduction but also restricts the free movement of anions through the spatial confinement effect of its chiral environment, which aligns with the “anion immobilization” design concept of single-ion conductors. For example, Du et al. modified polypropylene separators with ion-regulated hydroxypropyl methylcellulose CLC coatings. They found that metal cations (Na^+^, Li^+^, K^+^) can effectively tune the helical pitch of CLCs, with the largest pitch achieved under K^+^ regulation. The PP@HPK separator exhibited strong affinity for both PF_6_^−^ and Li^+^, delivering a high ***t***_Li_^+^ of 0.74 and stable cycling for 1530 and 560 cycles in LiFePO_4_ and NCM811 lithium metal full cells, respectively [13]. Lee et al. revealed that the ordered nanochannels in cholesteric liquid crystal cellulose nanocrystal films can guide uniform Li^+^ flux distribution and effectively suppress dendrite growth on lithium metal anodes [14]. Beyond the construction of ordered transport channels, molecular-level structural design has also been demonstrated as an effective strategy to regulate interfacial stability in electrochemical energy storage systems [15]. These works verify the unique potential of cholesteric helical structures in regulating ion transport.

Nevertheless, translating cholesteric liquid crystal structures into practically applicable free-standing electrolyte membranes still faces severe challenges. Traditional cholesteric liquid crystal polymer networks (CLCNs) require high crosslinking density to maintain helical order, resulting in brittle materials with extremely low elongation at break, which have difficulty withstanding the mechanical stress induced by electrode volume variation during LIB cycling [16]. This dilemma directs our attention to cholesteric liquid crystal elastomers (CLCEs)—materials that organically integrate the inherent helical supramolecular structure of cholesteric liquid crystals with the rubber elasticity of elastomers, achieving synergistic unity of microscopic ordered arrangement and macroscopic flexibility at the molecular level. The crosslinked elastic network endows the material with reversible stretch-recovery mechanical properties: the helical pitch changes reversibly under external force, and both the molecular ordered structure and macroscopic shape recover upon stress release. This overcomes the drawbacks of traditional cholesteric liquid crystals (incapable of deformation) and common elastomers (lacking well-defined ordered channels) [17,18]. In recent years, the exploration of liquid crystal elastomers (LCEs) in the field of LIB electrolytes has shown preliminary promise. For example, Yan et al. prepared novel solid electrolytes by combining LCEs with ionic liquids via UV-initiated copolymerization, which weakened lithium salt coordination and delivered a ***t***_Li_^+^ of 0.61. The lithium symmetric cells could cycle stably for 1000 h without obvious polarization rise [19]. Niu et al. introduced succinonitrile as a plasticizer into an LCE matrix to obtain LCE-SN electrolytes, which simultaneously achieved a mechanical strength of 0.52 MPa and a room-temperature ionic conductivity of 5.45 mS cm^−1^ and maintained stable long-term cycling when assembled into NCM811 high-voltage batteries [20]. However, systematic reports on free-standing membrane systems that integrate cholesteric liquid crystal elastomers with single-ion conductor design concepts remain absent, which constitutes the starting point of this work.

In our previous work [21], we coated liquid crystal polymer network-based single-ion conductors (denoted CLCN-Li) onto a flexible poly(vinylidene fluoride-co-hexafluoropropylene) (P(VDF-HFP)) support membrane, which improved the mechanical flexibility to a certain extent. Although a lithium-ion transference number as high as 0.98 was obtained, the presence of the host polymer in the composite system inevitably disrupted the long-range ordered arrangement of liquid crystal mesogens, impairing the oriented transport effect of the cholesteric helical structure. In this work, we designed and fabricated a series of CLCE free-standing single-ion conductive membranes without any support substrate or inorganic fillers, aiming to provide quasi-solid electrolytes with both high safety and excellent electrochemical performance for LIBs. By regulating the concentration of the chiral dopant, we systematically compared the effects of three pitches on Li^+^ transport kinetics. Meanwhile, to investigate the effect of helical chirality and dopant species, enantiomeric dopants S5011 (left-handed) and R5011 (right-handed), as well as right-handed chiral dopant CA-iso, were adopted to construct helical structures with different handedness and molecular packing states. The ionic conductivity does not follow a simple chirality-dependent rule and is jointly affected by helical pitch, molecular packing density and dopant structure. Remarkably, the membrane with the shortest pitch exhibits the highest lithium-ion transference number, which is attributed to denser molecular packing and an increased density of anionic anchoring sites per unit length. Furthermore, the addition of a small amount of exogenous lithium salt can synergistically enhance the ionic conductivity without compromising the high lithium-ion transference number. This work demonstrates that free-standing CLCE single-ion conductor membranes successfully circumvent the brittleness of CLCNs and the complexity of composite systems, providing an easy and effective design strategy for high-performance quasi-solid electrolytes.

## 2. Results and Discussion

**Figure 1:**
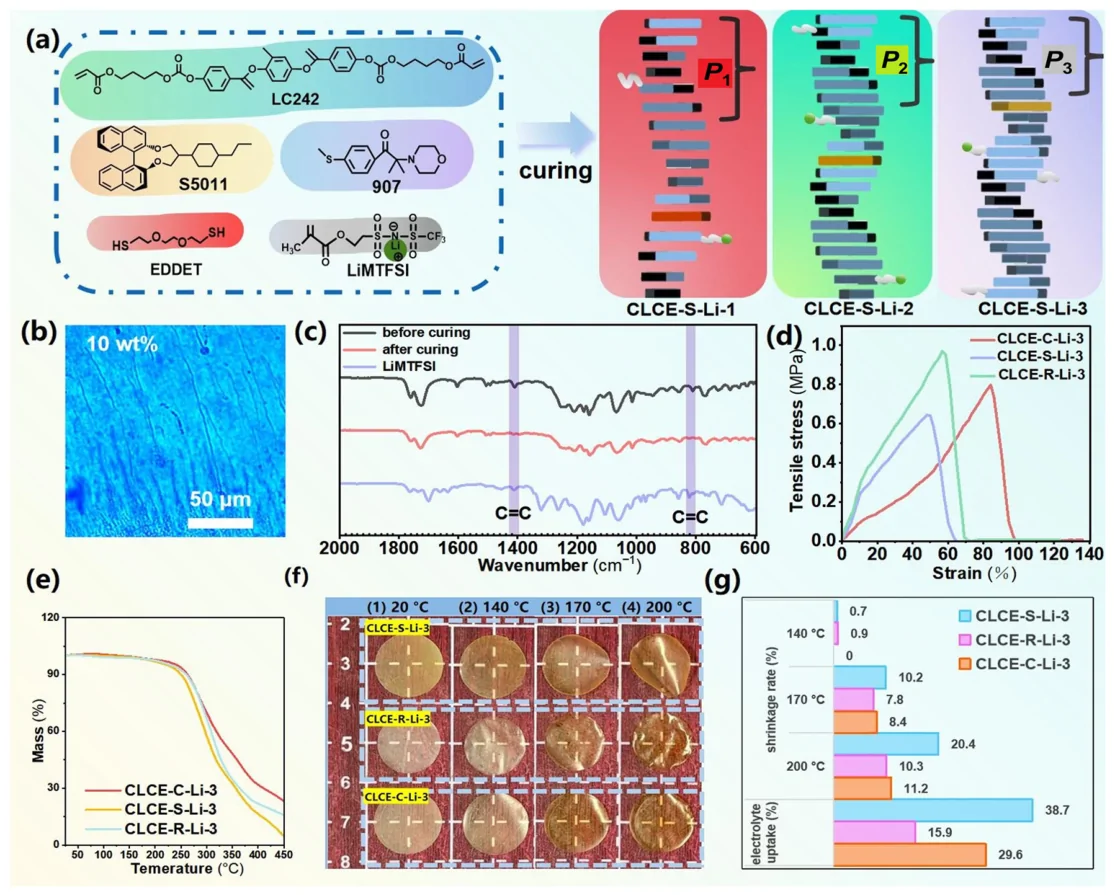
(**a**) Scheme of fabrication process of CLCE-S-Li films. (**b**) POM image of cholesteric liquid crystal mixture with LiMTFSI content of 10 wt% at 73.6 °C. (**c**) FT-IR spectra of the blend of LiMTFSI and cholesteric liquid crystal in both uncured and cured states. (**d**) Stress–strain curves of CLCE-S-Li-3, CLCE-R-Li-3 and CLCE-C-Li-3 films (collectively denoted as CLCE-X-Li-3 series). (**e**) TGA curves of CLCE-X-Li-3 series. (**f**) Thermal dimensional stability measured at (1) 20 °C, (2) 140 °C for 1 h, (3) 170 °C for 1 h and (4) 200 °C for 1 h. (**g**) The thermal shrinkage rate and electrolyte uptake of CLCE-X-Li-3 series.

Figure 1a illustrates the molecular structures of the raw materials and a schematic diagram of UV-induced curing fabrication. LC242 diacrylate and EDDET dithiol undergo thiol–ene click crosslinking, while chiral dopants induce the helical self-assembly of liquid crystal mesogens along a uniaxial direction. The pendant acrylic double bonds of LiMTFSI participate in the crosslinking reaction concurrently, such that TFSI^−^ anions are covalently anchored within the three-dimensional elastic network. The helical pitch of the CLCE films can be continuously tuned by adjusting the loading of the S5011 chiral dopant. Based on the cross-sectional FE-SEM images in Figure S1, the periodic cholesteric structures of the samples were observed and quantified, yielding helical pitch values of 411 nm (**P**_1_), 340 nm (**P**_2_) and 256 nm (**P**_3_) for the three samples, respectively, which follow a decreasing trend with rising dopant content. This result intuitively validates the dual modulation effect of both the type and doping concentration of chiral dopants on the helical pitch. The sample with the minimum pitch features a more compact molecular arrangement, a higher packing density of liquid crystal mesogens per unit length, and a larger number of oxygen coordination sites within the helical channels [22]. Figure 1b shows the POM micrograph of the 10 wt% LiMTFSI-doped thin film. Continuous, defect-free parallel cholesteric striation textures are visible across the entire field of view, with uniformly dispersed liquid crystal domains free of agglomeration [23,24]. Figure 1c presents the FT-IR spectra comparing the characteristic absorptions of the samples before and after curing. The uncured precursor exhibits characteristic peaks of acrylic C=C double bonds at 820 cm^−1^ and 1410 cm^−1^. After UV crosslinking, both absorption peaks disappear completely, indicating the consumption of acrylate groups during the curing process. The characteristic absorption peaks of LiMTFSI are preserved in the cured film, suggesting that the LiMTFSI moieties remain intact and are likely incorporated into the network. These spectral features are consistent with the formation of a crosslinked structure and the possible anchoring of the LiMTFSI monomer within the CLCE backbone.

Figure S2 shows the cross-sectional FE-SEM image (from which an overall film thickness of approximately 100 μm is determined) and the corresponding EDS elemental mapping images of the CLCE-C-Li-3 film. The four elements C, O, F and S are homogeneously distributed across the entire cross section, with no evident elemental enrichment or phase separation, verifying the uniform dispersion of each component within the elastic network. The stress–strain curves (Figure 1d) reveal tensile strengths of 0.66, 0.97, and 0.79 MPa for the CLCE-S-Li-3, CLCE-R-Li-3, and CLCE-C-Li-3 films, respectively, with corresponding elongation at break values of 64.8%, 71.2%, and 99.5%. These mechanical properties demonstrate satisfactory flexibility and ductility for all three films, which are essential for effectively accommodating the volume changes of the electrode during cycling and for maintaining stable interfacial contact between the electrode and the electrolyte [25,26]. The TGA curves (Figure 1e) reveal that the thermal decomposition temperatures of the CLCE-S-Li-3, CLCE-R-Li-3, and CLCE-C-Li-3 films are 223, 230, and 243 °C, respectively. These values, all well above the typical operating temperature range of lithium-ion batteries, demonstrate satisfactory thermal stability for all three electrolyte films, ensuring resistance against thermal degradation during operation or under abusive heating. As shown in Figure 1f,g, the CLCE-S-Li-3, CLCE-R-Li-3, and CLCE-C-Li-3 films all undergo thermal shrinkage when heated from room temperature to 200 °C, while their overall morphology remains intact, and the shrinkage is within a controllable range, indicating thermal shrinkage resistance and thermal stability for all three films. In addition, electrolyte uptake is observed for all three films, suggesting the presence of pore structures within the membranes, which facilitate infiltration and storage of the liquid electrolyte [27].

**Figure 2:**
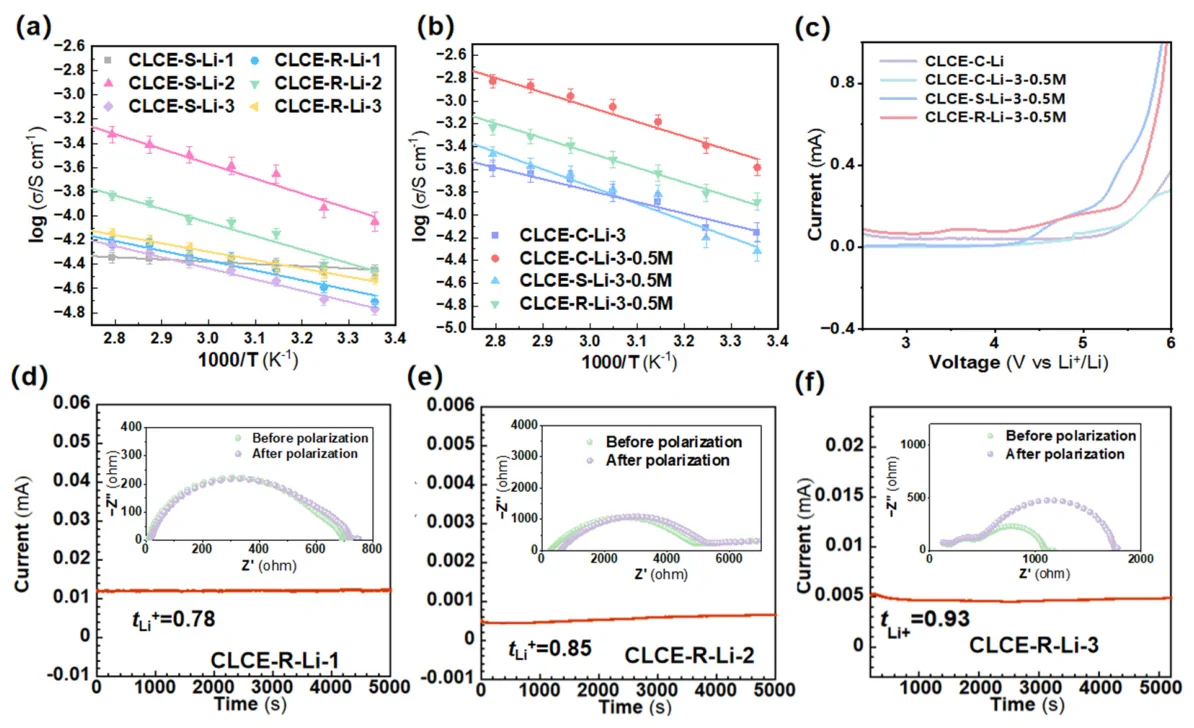
(**a**,**b**) Arrhenius plots of ionic conductivity for the as-prepared CLCE-based electrolyte membranes; (**c**) comparison of ionic conductivity, lithium-ion transference number and LSV of electrolyte membranes; (**d**–**f**) the chronoamperometry profiles during polarization of CLCE-S-Li electrolyte membranes; insets are the Nyquist impedance spectra measured under initial and steady-state current conditions.

The above obtained CLCE-Li films were immersed in plasticizer (EC/DMC mixed solution) to make corresponding quasi-solid electrolyte membranes. Their electrochemical performance is evaluated and shown in Figure 2 and Figures S3–S7, with the relevant data collected and listed in Table 1. The original impedance spectra for bulk resistance at various temperatures are provided in Figures S3 and S4. Figure 2a,b present the Arrhenius plots of ionic conductivity for all samples. The conductivity of each sample increases monotonically with rising temperature, consistent with the typical Arrhenius-type conduction behavior of polymer electrolytes [28,29,30]. At a similar helical pitch level, the CLCE-C-Li-3 (264 nm) exhibits a room temperature ionic conductivity of 7.0 × 10^−5^ S·cm^−1^, outperforming CLCE-S-Li-3 (256 nm) and CLCE-R-Li-3 (251 nm). When adding 0.5 M LiTFSI into the plasticizer to increase the amount of charge carrier, the room temperature conductivity of the obtained CLCE-C-Li-3-0.5M increases to 2.6 × 10^−4^ S·cm^−1^, representing the highest value among the series. The electrochemical stability window was evaluated by linear sweep voltammetry (Figure 2c and Figure S5). All samples exhibit oxidation stability potentials exceeding 4.2 V, with CLCE-C-Li-3-0.5M reaching 5.2 V, which is compatible with the operating voltages of mainstream cathodes such as LiFePO_4_. ***t***_Li_^+^ was determined by chronoamperometry combined with AC impedance measurements before and after polarization; the corresponding test curves are shown in Figure 2d–f, Figures S6 and S7. The ***t***_Li_^+^ exhibits an overall pitch-dependent regulation trend, with the smallest-pitch sample showing the highest value in each dopant system. The R-series presents a strictly monotonic increase, while the S-series shows a slight fluctuation at medium pitch due to secondary effects of dopant structure on molecular packing and anion distribution. Among them, the CLCE-C-Li-3 with the smallest pitch achieves a transference number of 0.94, approaching the level of an ideal single-ion conductor. This result is in full agreement with the core mechanism of this work: a smaller pitch not only increases the density of covalently anchored anionic sites within the helical channel and enhances spatial confinement, thereby effectively suppressing anion migration, but also provides a higher density of lithium-coordinating sites (such as carbonyl and ether oxygen groups) per unit length, facilitating more efficient directional relay transport of lithium ions along the helical pathway and thus further elevating the contribution of lithium ions to the total charge-carrier flux. After the introduction of free lithium salt into plasticizer, the ***t***_Li_^+^ remains in the range of 0.71–0.84, still within the regime of near-single-ion conduction, which is reasonably ascribed to the contribution of the cholesteric structure. This enables a favorable balance between high transference number and ionic conductivity, simultaneously ensuring efficient ion transport. 

**Figure 3:**
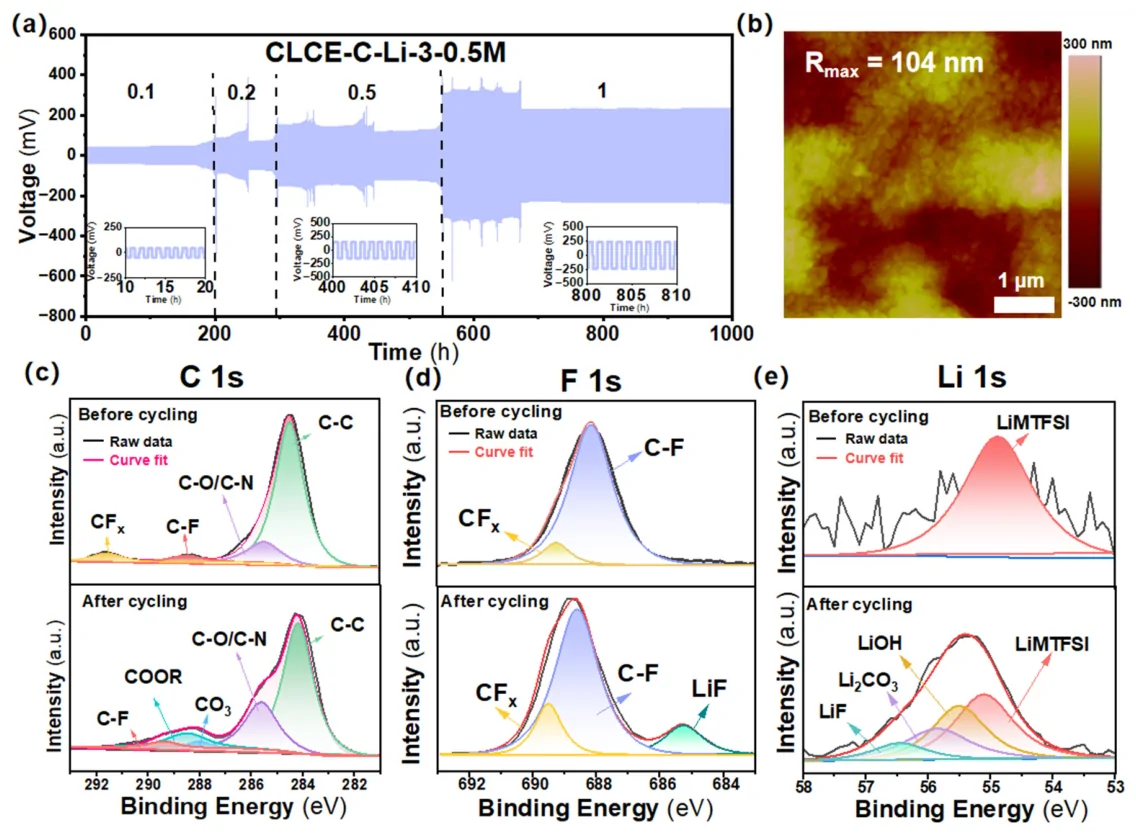
(**a**) The voltage curves of CLCE-C-Li-3-0.5M membrane and the corresponding partial voltage enlarged graphs at current densities of 0.1, 0.2, 0.5 and 1 mA cm^−2^; (**b**) AFM images of Li metal electrode; (**c**–**e**) XPS spectra of CLCE-C-Li-3-0.5M membrane in Li|CLCE-C-0Li-3-0.5M|Li battery after cycling.

Figure 3a presents the galvanostatic charge–discharge profiles of Li symmetric cells assembled with the CLCE-C-Li-3-0.5M electrolyte. Under gradient current densities ranging from 0.1 to 1 mA cm^−2^, the cells maintain steady polarization voltages without abrupt voltage rise or short-circuit failure over 1000 h of prolonged cycling, indicating favorable interfacial stability throughout the operation. After cycling, the cells were disassembled for post-mortem characterization. AFM observation of the cycled lithium metal surface (Figure 3b) reveals a flat and dense morphology with a maximum surface roughness of only 104 nm, and no conspicuous dendrite protrusions are detected. This result verifies that the ordered helical channels can homogenize lithium-ion flux and guide uniform lithium plating. XPS compositional analysis of the cycled electrolyte membrane (Figure 3c–e) shows that a pronounced LiF characteristic peak appears in the F 1s spectrum after cycling; typical solid electrolyte interphase (SEI) components including LiF and Li_2_CO_3_ are simultaneously identified in the Li 1s spectrum. These findings suggest that the electrolyte undergoes moderate reductive decomposition on the lithium surface, forming a stable LiF-rich solid electrolyte interphase layer.

**Figure 4:**
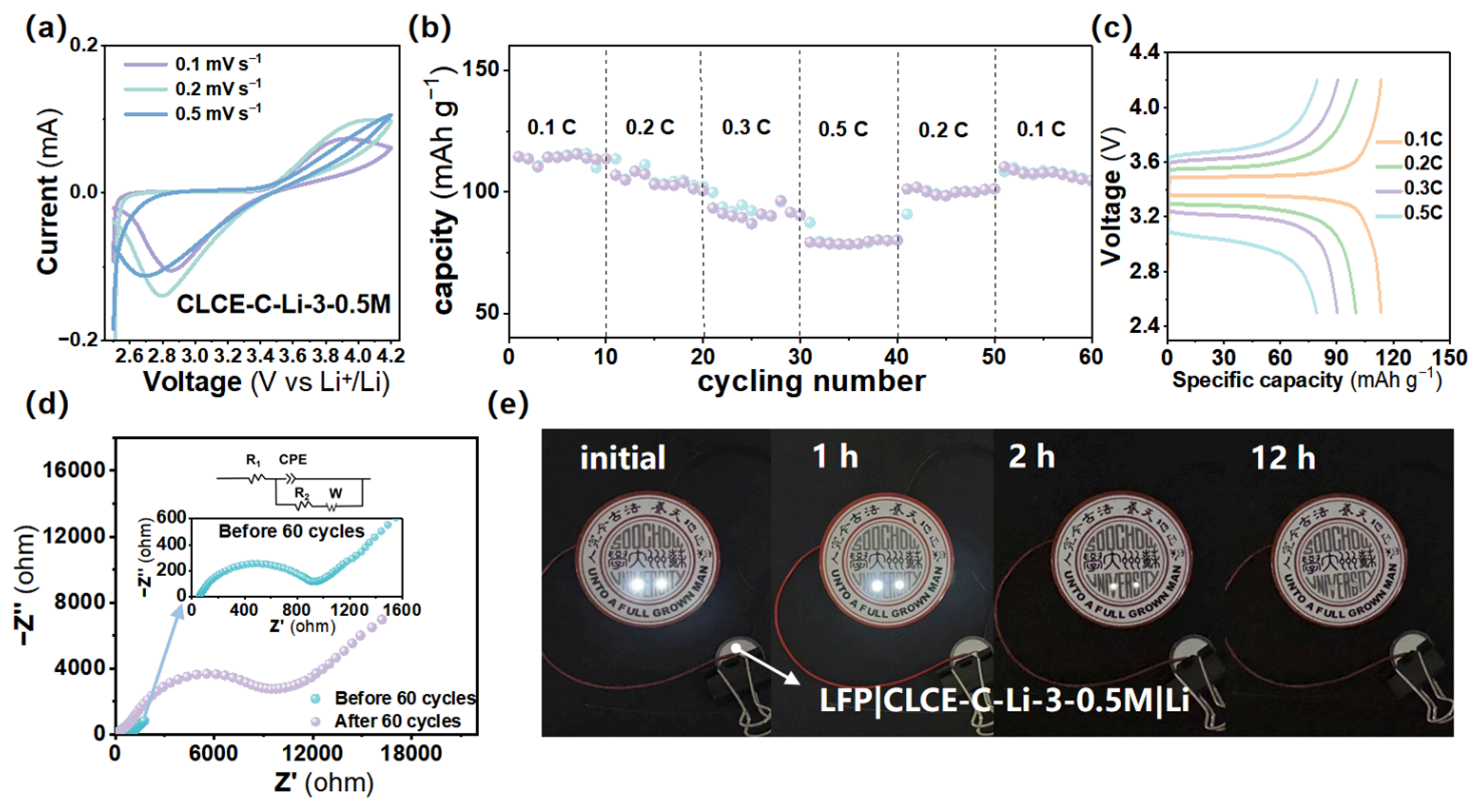
LFP|CLCE-C-Li-3-0.5M|Li battery: (**a**) cyclic voltammetry curves at 0.1, 0.2 and 0.5 mV s^−1^; (**b**) galvanostatic charge capacity at various current densities from 0.1, 0.2, 0.3 and 0.5 C (1C = 170 mA h g^−1^); (**c**) the first charge–discharge curves at 0.1, 0.2, 0.3 and 0.5 C; (**d**) Nyquist diagram before and after 60 cycles (the inset is the equivalent circuit model); (**e**) LED lamps lit up by LFP|CLCE-C-Li-3-0.5M|Li battery.

To evaluate the practical application potential of the as-prepared electrolyte, quasi-solid LiFePO_4_||Li full cells were assembled using CLCE-C-Li-3-0.5M as the electrolyte membrane. The cyclic voltammetry curves in Figure 4a display a pair of well-defined symmetric redox peaks around 3.4 V, corresponding to the lithium deintercalation and intercalation process of LiFePO_4_. The absence of miscellaneous peaks indicates favorable compatibility between the electrolyte and the cathode, along with excellent electrochemical reaction reversibility [31,32]. The rate capability is illustrated in Figure 4b,c. The cell delivers a specific discharge capacity of 119.0 mAh g^−1^ at 0.1 C. When the current density rises to 0.2 C, 0.3 C and 0.5 C, the specific discharge capacities reach 113.5, 100.5 and 90.3 mAh g^−1^, respectively, showing mild capacity fading. Slight capacity fluctuations during rate switching arise from synergistic gradual cell activation and rate-dependent polarization. Repeated Li^+^ insertion–extraction optimizes the electrolyte–cathode interface at low rates, while a high current induces strong polarization owing to limited Li^+^ transport in CLCE electrolyte, leading to transient capacity drop [33,34]. As the current density is stepwise restored to 0.2 C and 0.1 C, the specific discharge capacities recover to 112.9 and 117.8 mAh g^−1^, respectively, suggesting excellent capacity recovery and outstanding rate reversibility. As presented in Figure 4d with the corresponding equivalent circuit in the inset, each Nyquist plot consists of a high-frequency semicircle reflecting interfacial charge transfer resistance and a low-frequency Warburg slope related to lithium-ion diffusion. The cell shows a low initial charge transfer resistance of ~800 Ω, which rises to around 12 kΩ after 60 cycles. This increase mainly stems from the formation and thickening of the SEI film on the lithium anode. Nevertheless, the impedance shows no uncontrolled surge, indicating the interface gradually stabilizes after initial SEI evolution without severe persistent parasitic reactions [35,36]. The long-term cycling stability of the LiFePO_4_||Li full cell was further evaluated at 0.2 C for 80 cycles (Figure S8a). The cell exhibits a slight capacity decay during the initial activation stage, after which the discharge capacity stabilizes at ~80 mAh g^−1^ with an average Coulombic efficiency above 99%, indicating good cycling reversibility of the system. By comparing the EIS before and after 80 cycles (Figure S8b), it is found that the charge-transfer resistance shows a moderate increase after long-term cycling, which is mainly attributed to the formation of the SEI layer in the initial cycling stage. The impedance growth rate gradually decreases with cycling, indicating that the electrode–electrolyte interface tends to be stable after the formation of a robust interphase layer, which further verifies the favorable interfacial compatibility of the CLCE electrolyte. The assembled cell can steadily power a light-emitting diode (LED) indicator (Figure 4e), which visually validates its practical operation feasibility.

**Figure 5:**
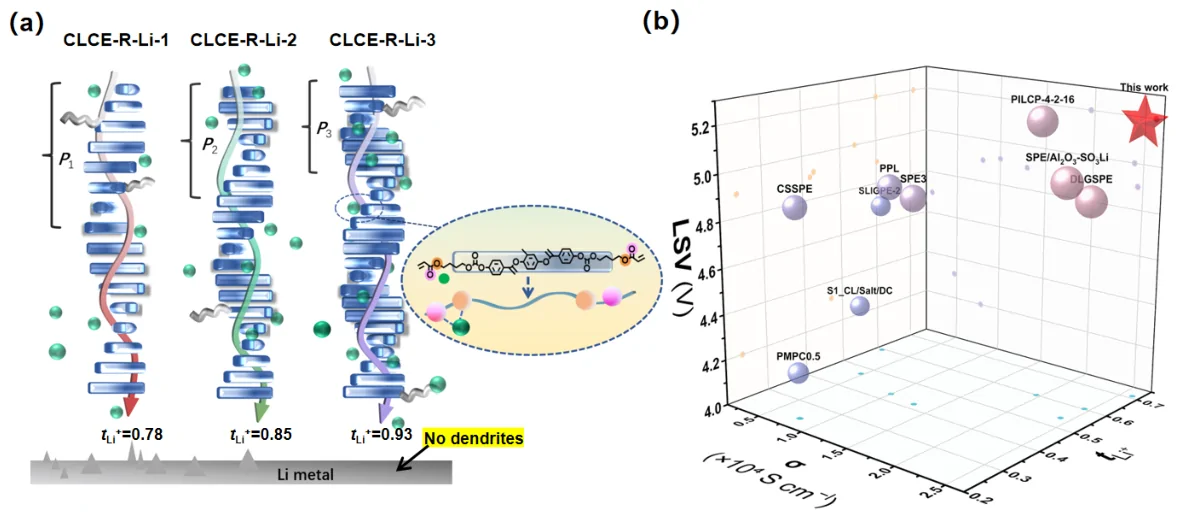
(**a**) Schematic diagram of the Li ion transport in CLCE; (**b**) 3D scatter plot comparing the ionic conductivity, lithium-ion transference number and electrochemical stability window of the as-prepared electrolyte membrane with representative polymer electrolyte membranes reported in the literatures, including PILCP-4-2-16 [37], CSSPE [38], SPE3 [39], S1_CL/Salt/DC [40], PMPC0.5 [41], SLIGPE-2 [42], PPL [43], SPE/Al2O3-SO3Li [44], and DLGSPE [45].

Figure 5a illustrates the synergistic ion transport mechanism of the CLCE single-ion conductor. The excellent comprehensive electrochemical performance of the electrolyte mainly stems from the following four synergistic contributions. (1) The self-assembled cholesteric liquid crystals construct directional conduction pathways for ion transport. The densely distributed carbonyl and ether oxygen groups on the inner walls of the channels act as reversible coordination sites for lithium ions, driving ions to migrate directionally along the helical axis in a relay mode and effectively reducing the transport resistance caused by disordered diffusion. (2) TFSI^−^ anions are covalently grafted onto the polymer backbone via LiMTFSI monomers, which eliminates the cation–anion drag effect at the molecular level, significantly increases the lithium-ion transference number, and fundamentally alleviates concentration polarization during battery cycling. (3) The introduction of a small amount of additional lithium salt into the plasticized system effectively raises the free carrier concentration. While preserving the near-single-ion conductive property of the electrolyte, it strongly improves room-temperature ion conduction efficiency, achieving a well-balanced performance between the transference number and ionic conductivity. (4) The well-ordered helical channels homogenize the lithium-ion flux and induce the in situ formation of a stable high-modulus LiF-rich interphase layer on the lithium anode surface. This structure ensures efficient ion shuttling while suppressing dendrite growth, enhancing the interfacial stability and safety over long-term cycling. Benefiting from the synergistic optimization of the above mechanisms, the three-dimensional performance comparison with reported analogous polymer electrolytes in Figure 5b [37,38,39,40,41,42,43,44,45] confirms that the optimal sample in this work delivers more competitive overall performance in ionic conductivity, lithium-ion transference number and electrochemical stability window, fully validating the rationality and application potential of this design strategy.

## 3. Materials and Methods

### 3.1. Materials

Tetrahydrofuran (THF, A.R., Lingfeng, Shanghai, China), N,N-dimethylformamide (DMF, 99%, Qiangsheng, Shanghai, China), dichloromethane (A.R., Qiangsheng, Shanghai, China), cyclohexanone (A.R., Yonghua, Suzhou, China), ethyl acetate (EA, A.R., Yonghua, Suzhou, China), ethylene carbonate/dimethyl carbonate (EC/DMC, 1:1, *v*/*v*, DoDoChem, Suzhou, China), anhydrous magnesium sulfate (99.0%, Macklin, Shanghai, China), lithium hydride (97.0%, Aladdin, Shanghai, China), triethylamine (TEA, 99.5%, Merck, Shanghai, China), chlorosulfonic acid (A.R., Qiangsheng, Shanghai, China), trifluoromethanesulfonamide (98%, Aladdin, Shanghai, China), potassium 3-(methylacryloxy)propane-1-sulfonic acid (A.R., Aladdin, Shanghai, China), 2,2′-(ethylenedioxy)diethanethiol (EDDET, 99%, Aladdin, Shanghai, China), photoinitiator 2-methyl-1-(4-methylthiophenyl)-2-morpholinopropan-1-one (Irgacure907, 98%, Aladdin, Shanghai, China), nematic liquid crystal monomer 4-(((4-acryloyloxybutoxy)carbonyl)oxy) benzoic acid-2-methyl-1,4-diphenolester (LC242, 98%, Chuangtuo, Nanjing, China), polytetrafluoroethylene (PTFE, Dezhou Hongyu, Dezhou, China), LiFePO4 (LFP, 99%, Macklin, Shanghai, China), R5011 (99%, Shanghai Sanjiang New Materials Technology Co., Ltd., Shanghai, China), S5011 (99%, Shanghai Sanjiang New Materials Technology Co., Ltd., Shanghai, China), and CA-iso (99%, Shanghai Sanjiang New Materials Technology Co., Ltd., Shanghai, China) were purchased.

### 3.2. Fabrication of CLCE-Li Membranes

Li salt LiMTFSI was prepared according to the literature (in the Supporting Materials [46]). LC242, CA-iso and EDDET were mixed in TEA/THF mixed solvent at the ratios listed in Table S1 under stirring to obtain liquid crystal mixtures. The mixtures were placed in an oil bath and reacted isothermally at 35 °C for 24 h to generate oligomers. The as-obtained oligomers were dissolved in EA and washed twice with dilute hydrochloric acid and once with deionized water sequentially. After suction filtration and rotary evaporation, a pale yellow oily substance was acquired. Excess ethanol was added to the oily product and stirred for 15 min. After the ethanol was removed, the residue was dissolved in 10 g cyclohexane to yield the target liquid crystal mixture. Then, 2 g of cholesteric liquid crystal oligomer was weighed, followed by the addition of 3 wt% photoinitiator Irg907 and 10 wt% LiMTFSI. A mixed solvent of cyclohexanone and EA (volume ratio = 4:1) was then supplemented to prepare a casting solution with a solid content of 20 wt%. This solution was cast onto pre-oriented polyethylene terephthalate (PET) substrates and photocured under ultraviolet (UV) irradiation to obtain CLCE-C-Li-3 films with ordered layer stacking structures, where “3” refers to the pitch around 264 nm. 

The above procedures were repeated using S5011 and R5011 as the chiral dopant, respectively. By tuning the dopant loading to regulate the helical pitch, three left-handed stacking CLCE-S-Li films with gradient pitches (CLCE-S-Li-1: 411 nm, CLCE-S-Li-2: 340 nm, CLCE-S-Li-3: 256 nm) and three right-handed stacking CLCE-R-Li films (CLCE-R-Li-1: 407 nm, CLCE-R-Li-2: 321 nm, CLCE-R-Li-3: 251 nm) were successfully fabricated.

### 3.3. Methods and Characterizations

Temperature-dependent polarized optical microscopy (POM) characterizations were implemented on a microscope manufactured by Leica Microsystems CMS GmbH (Leica Microsystems GmbH, Mannheim, Germany), which was coupled with a Linkam LTS420 heating stage to realize thermal control during observation. Cross-section samples for field emission scanning electron microscopy (FE-SEM) testing were pre-fractured via liquid nitrogen freezing to acquire intact fracture surfaces, and all FE-SEM micrographs were collected on a Hitachi S-4800 device (Hitachi Ltd., Tokyo, Japan) under a 5.0 kV accelerating voltage. Thermogravimetric analysis (TGA) measurements were completed on a Thermal Analysis TG/TGA 6300 analyzer (SII NanoTechnology Inc., Tokyo, Japan). To eliminate sample oxidation throughout the test, the whole measurement process was carried out under continuous nitrogen flow at a constant heating ramp of 10 °C per minute. The elemental chemical composition of specimens was quantified through elemental analysis using a Vario EL III elemental analyzer (Elementar Analysensysteme GmbH, Frankfurt, Germany). A high-pressure mercury lamp model MINHIO 4012-20 (1000 W input power, MINHIO Intelligent Equipment Co., Ltd., Shenzhen, China) was adopted to conduct photopolymerization related experiments. Fourier transform infrared (FT-IR) spectra were acquired on a VRETEX 70 spectrometer (Bruker Optics GmbH & Co. KG, Ettlingen, Germany) with a fixed spectral resolution of 4.0 cm^−1^; a total of 16 repeated scanning cycles were averaged for each sample to improve the signal-to-noise performance of spectral data. Detailed procedures and calculation methods of electrochemical tests are provided in the Supporting Information.

## 4. Conclusions

In summary, this work combines the helical ordered structure of cholesteric liquid crystal elastomers with the design concept of single-ion conductors and successfully constructs a series of substrate-free quasi-solid electrolyte membranes with tunable helical pitches and chiral dopants. The overall regulation law of helical pitch on ion transport is clarified: the lithium-ion transference number generally increases with the decrease in pitch. The CA-iso-doped CLCE-C-Li-3 membrane delivers a high transference number of 0.94, and its plasticized sample achieves a room-temperature ionic conductivity of 2.6 × 10^−4^ S·cm^−1^. The ordered helical channels homogenize lithium-ion flux and induce the formation of a stable LiF-rich interphase on lithium metal, with a maximum surface roughness of only 104 nm after cycling. The assembled Li symmetric cell maintains stable cycling for 1000 h, and the LiFePO_4_ full cell exhibits favorable rate performance and reversibility. This work provides a feasible strategy for the structural optimization of ordered-transport quasi-solid electrolytes.

## Figures and Tables

**Table 1:** Ionic conductivity, lithium-ion transference number and LSV of electrolyte membranes.

Sample	Pitch (nm)	LiTFSI in Plasticizer	σ (S cm^−1^)	*t*_Li_^+^	LSV (V)
CLCE-S-Li-1	411	-	3.6 × 10^−5^	0.84	4.5
CLCE-R-Li-1	407	-	2.0 × 10^−5^	0.78	4.9
CLCE-S-Li-2	340	-	9.1 × 10^−5^	0.78	5.1
CLCE-R-Li-2	321	-	3.4 × 10^−5^	0.85	5.2
CLCE-S-Li-3	256	-	1.7 × 10^−5^	0.92	4.5
CLCE-R-Li-3	251	-	3.0 × 10^−5^	0.93	4.4
CLCE-C-Li-3	264	-	7.0 × 10^−5^	0.94	5.4
CLCE-S-Li-3-0.5M	256	0.5M ^#^	4.8 × 10^−5^	0.82	4.3
CLCE-R-Li-3-0.5M	251	0.5M	1.3 × 10^−4^	0.84	4.2
CLCE-C-Li-3-0.5M	264	0.5M	2.6 × 10^−4^	0.71	5.2

^#^: 0.5M represents 0.5 mol of LiTFSI dissolved in 1 L of plasticizer.

## Data Availability

The data presented in this study are available in the Supplementary Materials.

## Supplementary Materials

The following supporting information can be downloaded at https://www.mdpi.com/article/10.3390/molecules31162879/s1: Figure S1. Corresponding FE-SEM cross-sectional images, revealing the pitch parameters of their cholesteric liquid crystal structures. Figure S2. (a) FE-SEM and (b–e) corresponding EDS mapping images of CLCE-C-Li-3 composite film. Figures S3 and S4. Bulk resistances at different temperatures of electrolyte membranes. Figure S5. LSV profiles of electrolyte membranes. Figures S6 and S7. The chronoamperometry profiles during polarization, where insets are the Nyquist impedance spectra measured under initial and steady-state current conditions. Figure S8. LFP|CLCE-C-Li-3-0.5M|Li battery: (a) Cycling performance at 0.2 C; (b) Nyquist diagram before and after 80 cycles (the inset is the equivalent circuit model). Table S1. Raw material compositions for three liquid crystal oligomers. Table S2. Summary of key test conditions for referenced electrolyte systems in Figure 5b.
