# Supplementary material for: Cholesteric Liquid Crystal Elastomer-Based Single-Ion Conductor for Advanced Quasi-Solid Electrolyte Membranes
Source: Molecules. 2026 Aug 18;31(16):2879. doi: 10.3390/molecules31162879 (PMC13515214; doi:10.3390/molecules31162879)
Supplement: Supplementary file 1 [file molecules-31-02879-s001.zip › molecules-4465335-supplementary.pdf]

Cholesteric Liquid Crystal Elastomer-Based Single-Ion Conductor for Advanced Quasi-Solid Electrolyte Membranes

Tangqi Hu, Junxian Fu, Yonggang Yang and Yi Li *

Jiangsu Key Laboratory of Advanced Functional Polymer Materials, Department of Polymer Science and Engineering, College of Chemistry, Chemical Engineering and Materials Science, Soochow University, Suzhou 215123, China

* Correspondence: liyi@suda.edu.cn; Tel.: +86-512-65882052

Experimental Section

1. Synthesis of LiMTFSI

The LiMTFSI monomer was synthesized via a modified three-step route based on a reported method [46]. First, anhydrous potassium 3-(methacryloyloxy)propane-1-sulfonate was dispersed in THF with dimethylformamide as the catalyst under nitrogen atmosphere. After cooling to 0–1 °C, thionyl chloride was added dropwise under stirring. The mixture reacted at 0–1 °C for 1 h and then at room temperature for 12 h. The crude product was poured into ice water, extracted with dichloromethane, washed with deionized water, dried over anhydrous $MgSO_4$ and concentrated by rotary evaporation to obtain the sulfonyl chloride intermediate. Subsequently, the intermediate dissolved in anhydrous THF was added dropwise to an ice-cooled THF solution containing trifluoromethanesulfonamide and anhydrous triethylamine under nitrogen. After 1 h of reaction at 0 °C followed by 1 h of stirring at room temperature, the precipitate was removed by filtration. The filtrate was purified via extraction, washing, drying and evaporation to yield the sulfonimide intermediate. Finally, the above product was dissolved in anhydrous THF, and a LiH suspension in THF was added dropwise at 0 °C under inert atmosphere. After 2 h of reaction at room temperature, unreacted LiH was filtered off. The filtrate was washed

with hexane, concentrated and recrystallized from anhydrous dichloromethane to afford white crystalline LiMTFSI.

[46] Porcarelli, L., Shaplov, A. S., Salsamendi, M., Nair, J. R., Vygodskii, Y. S., Mecerreyes, D., Gerbaldi, C. Single-Ion Block Copoly(ionic liquid)s as Electrolytes for All-Solid State Lithium Batteries. *ACS Appl. Mater. Interfaces* **2016**, 8, 10350–10359.

2. Battery Assembly and Electrochemical Tests

Temperature-variable electrochemical impedance spectroscopy (EIS) was measured with a 5 mV AC signal, with test frequencies ranging from 100 kHz to 10 MHz.Linear sweep voltammetry (LSV) was scanned at 5.0 mV s^{-1}, and the test potential ranged from open-circuit voltage to 7.0 V (vs. Li/Li^{+}).All above electrochemical characterizations relied on CHI660E electrochemical workstation (Shanghai Chenhua Instrument Co., Ltd., Shanghai, China).Two LAND battery testers were adopted for galvanostatic cycling experiments. The CT3002A system from Wuhan Bluetooth Electronics Co., Ltd. was used for routine constant current charge/discharge tests.Symmetric Li/Li cells were cycled on CT2001A equipment at room temperature to investigate the interfacial matching property between lithium metal electrode and gel electrolyte film. All CV and charge-discharge cycle tests were performed between 2.5 V and 4.2 V. Room-temperature cycling tests with different current densities were conducted, with 1 C set to 170 mAh g^{-1}. CR2016 coin cells were assembled in an argon-protected glove box. The liquid electrolyte was 0.2 M LiTFSI dissolved in EC/DMC (1:1 by volume). The cathode slurry consisted of $LiFePO_4$, acetylene black and PVDF (mass ratio 8:1:1) dissolved in NMP. After coating on aluminum foil and overnight drying, the electrode was cut into 14 mm circular sheets with 1.8 mg active material. Lithium metal foil was used as the counter electrode, and 16 mm electrolyte film discs were used as separators. The $LiFePO_4$ cathode was prepared with an active material loading of ~ 1.6 mg cm^{-2}.

3. Calculations

SS|CLCE-Li|SS symmetric cells were assembled for impedance spectroscopy tests to obtain the ionic conductivity (σ) of membrane samples. The ionic conductivity was calculated via the following formula:

$$\sigma = \frac{1}{R_b S} \qquad (S1)$$

where R_b represents the bulk resistance of the electrolyte membrane, L stands for membrane thickness, and S corresponds to the contact area between electrode and electrolyte.

Li|CLCE-Li|Li symmetric cells were applied to measure the room-temperature t_{Li}^{+}. The testing method combined chronoamperometry and alternating current impedance spectroscopy. The value of t_{Li}^{+} was solved by the equation shown below:

$$t_{Li^+} = \frac{I_s(\Delta V - I_0 R_0)}{I_0(\Delta V - I_s R_s)} \qquad (S2)$$

An 80 mV polarization voltage was loaded on the cell during chronoamperometry, and the initial current I_0 as well as steady-state current I_s were recorded. AC impedance tests were implemented before and after polarization to acquire the initial interfacial resistance R_0 and steady-state interfacial resistance R_s.

Electrolyte uptake (%) of composite films was determined by the formula below:

$$\text{Electrolyte uptake (\%)} = \frac{M_2 - M_1}{M_1} \times 100\% \qquad (S3)$$

M_1 and M_2 correspond to the membrane weight before and after immersion in electrolyte, respectively.

Table S1. Raw material compositions for three liquid crystal oligomers

	**LC242 (g)**	**CA-iso (g)**	**EDDET (g)**
CLCE-C-Li-3	10.00	0.47	2.41
	**LC242 (g)**	**S5011 (g)**	**EDDET (g)**
CLCE-S-Li-1	9.49	0.21	2.40
CLCE-S-Li-2	9.44	0.25	2.40
CLCE-S-Li-3	9.41	0.29	2.40
	**LC242 (g)**	**R5011 (g)**	**EDDET (g)**
CLCE-R-Li-1	9.49	0.21	2.40
CLCE-R-Li-2	9.44	0.25	2.40
CLCE-R-Li-3	9.41	0.29	2.40

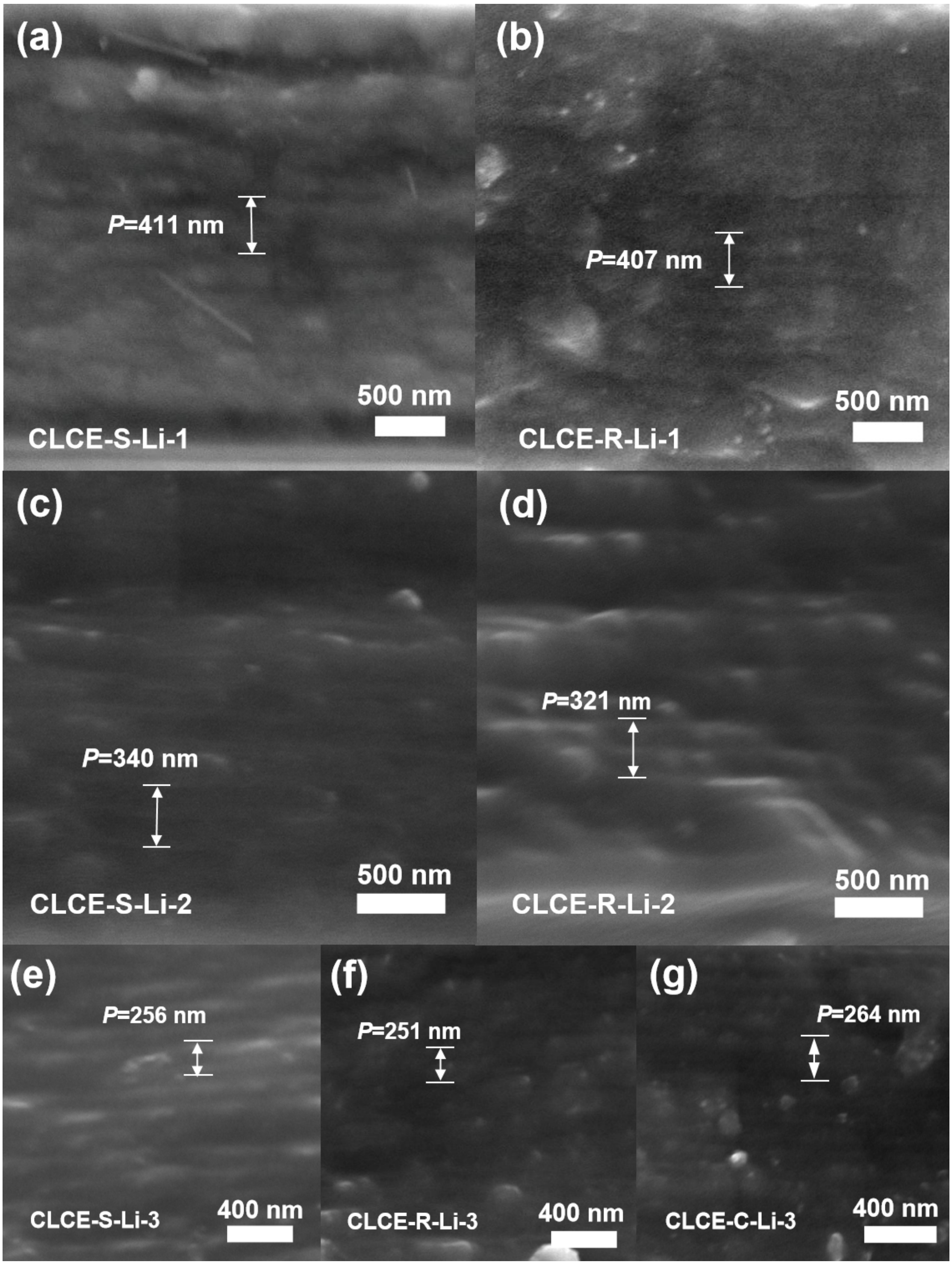

Figure S1. Corresponding FE-SEM cross-sectional images, revealing the pitch parameters of their cholesteric liquid crystal structures.

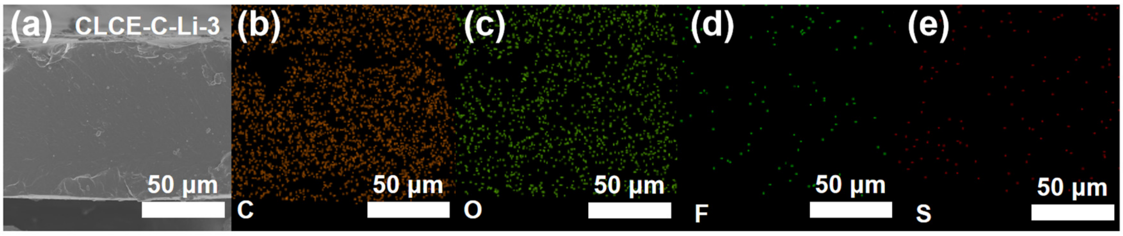

Figure S2. (a) FE-SEM and (b-e) corresponding EDS mapping images of CLCE-C-Li-3 composite film.

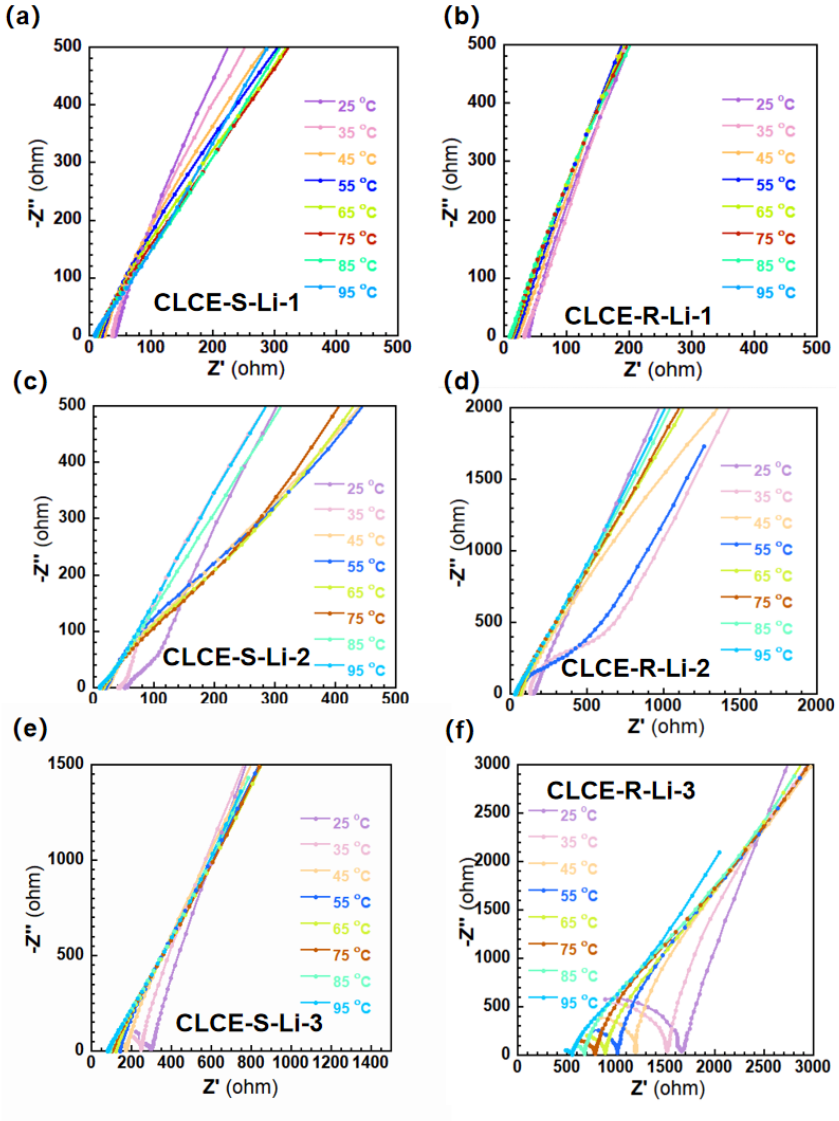

Figure S3. Bulk resistances at different temperatures of electrolyte membranes.

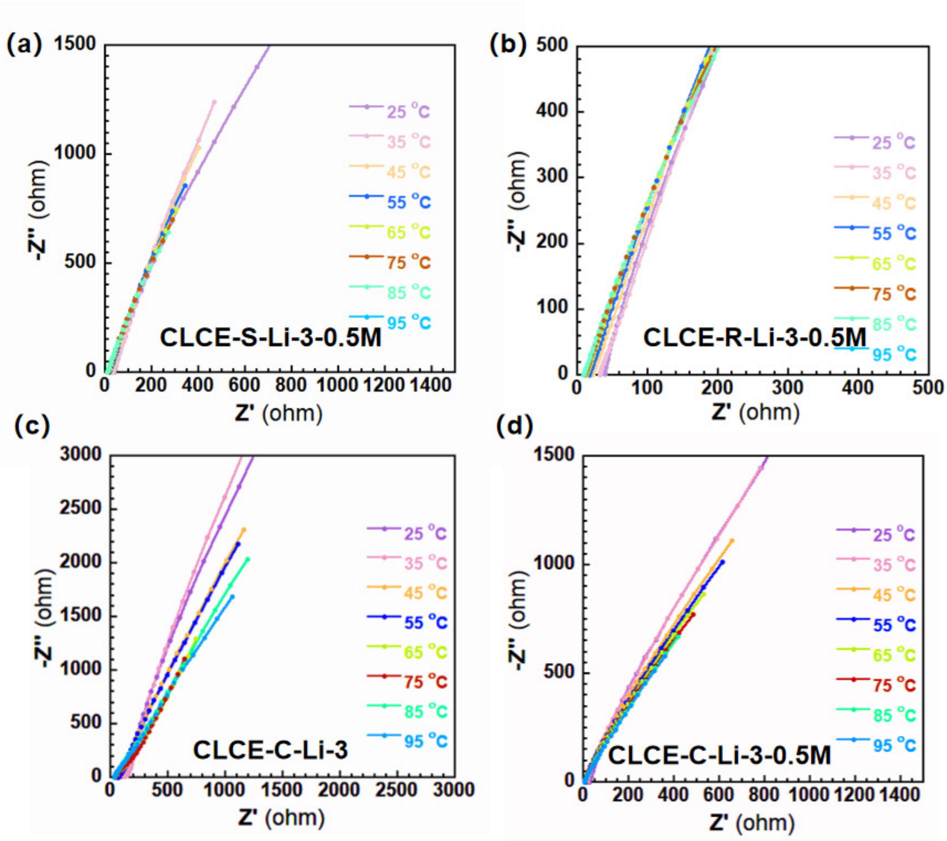

Figure S4. Bulk resistances at different temperatures of electrolyte membranes.

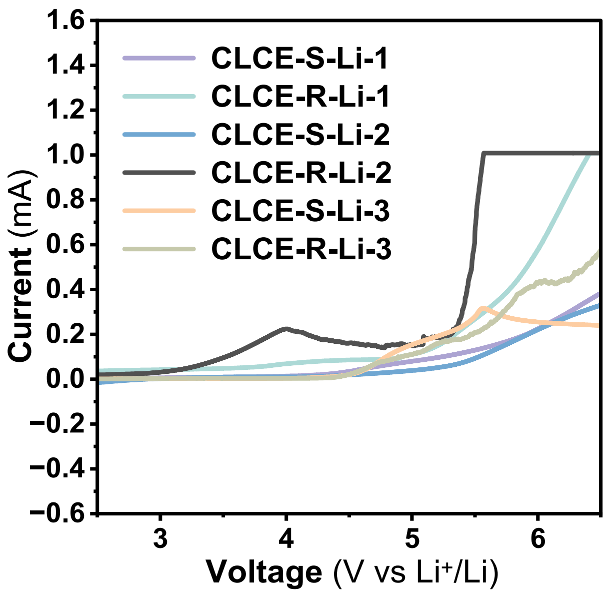

Figure S5. LSV profiles of electrolyte membranes.

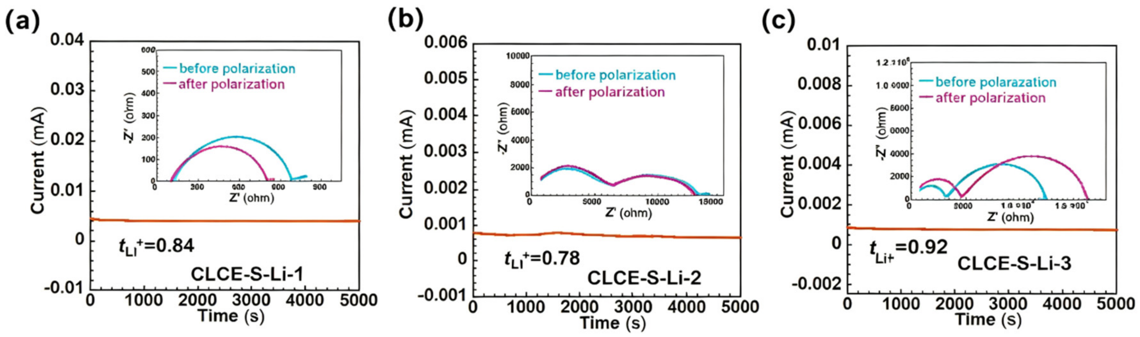

Figure S6. The chronoamperometry profiles during polarization, where insets are the Nyquist impedance spectra measured under initial and steady-state current conditions.

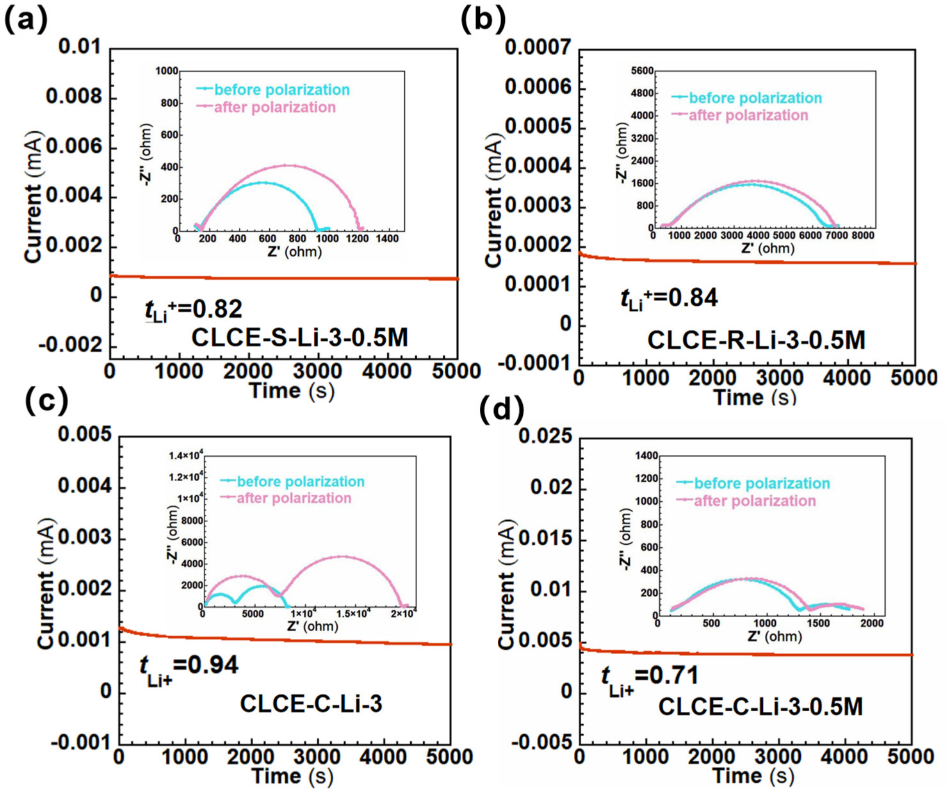

Figure S7. The chronoamperometry profiles during polarization, where insets are the Nyquist impedance spectra measured under initial and steady-state current conditions.

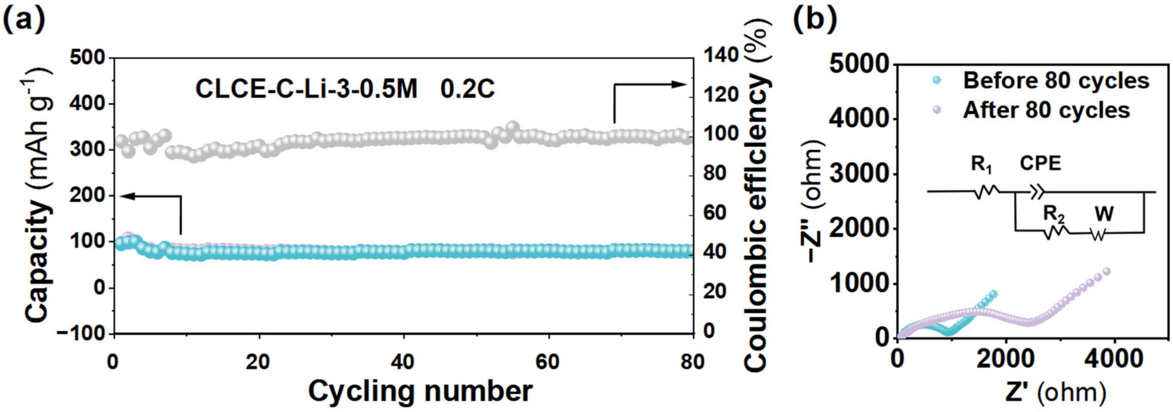

Figure S8. LFP|CLCE-C-Li-3-0.5M|Li battery: (a) Cycling performance at 0.2 C; (b) Nyquist diagram before and after 80 cycles (the inset is the equivalent circuit model).

Table S2. Summary of key test conditions for referenced electrolyte systems in Figure 5b.

Reference	Membrane Thickness (μm)	Test Temperature (°C)	Lithium Salt	Salt Concentration / Ratio
This work	~100	25	LiMTFSI	10 wt%
PILCP-4-2-16 [37]	~200	30	$LiBF_4$	[EO]/[Li] = 7
CSSPE [38]	~100	30	LiTFSI	30 wt% (polymer : salt = 1 : 0.3)
SPE3 [39]	~400	~25	LiTFSI	—
S1_CL/Salt/DC [40]	—	60	LiTFSI	—
PMPC0.5 [41]	—	25	$LiBF_4$	PMOBIm-BF_4 : PEGDA : $[C_{16}mim]BF_4$ = 3 : 1 : 0.5
SLIGPE-3.5 [42]	—	~25	LiSTFSI	PEGDMA : LiSTFSI = 1 : 3.5 (molar ratio)
PPL [43]	~22.4	30	LiTFSI	—
SPE/Al_2O_3-SO_3Li [44]	13	25	LiTFSI	—
DLGSPE [45]	200–240	30	LiTFSI	20 wt%

37. Wang, S.; Liu, X.; Wang, A.; Wang, Z.; Chen, J.; Zeng, Q.; Wang, X.; Zhang, L. An ionic liquid crystal-based solid polymer electrolyte with desirable ion-conducting channels for superior performance ambient-temperature lithium batteries. *Polym. Chem.* **2018**, 9, 4674–4682.
38. Wang, S.; Zhang, L.; Zeng, Q.; Liu, X.; Lai, W.-Y. Cellulose Microcrystals with Brush-Like Architectures as Flexible All-Solid-State Polymer Electrolyte. *ACS Sustainable Chem. Eng.* **2020**, 8, 3200–3207.
39. Li, J.; Huo, F.; Yang, Y.; Chen, T.; Cui, Y.; Cai, Y.; Zhang, H. Constructing stable lithium interfaces via coordination of fluorinated ether and liquid crystal for room-temperature solid-state lithium metal batteries. *Chem. Eng. J.* **2022**, 433, 133562.
40. Moisés, I. A.; Król, M.; Keus, G.; He, Z.; Innocenti, A.; Passerini, S.; Ruokolainen, J.; Gohy, J.-F. Enhancement of Lithium-Ion Conductivity in Liquid Crystalline Block Copolymer by Electric Field Alignment. *J. Am. Chem. Soc.* **2025**, 147, 20347–20358.
41. Chen, X.; Xie, Y.; Ling, Y.; Zhao, J.; Xu, Y.; Tong, Y.; Li, S.; Wang, Y. Ionic liquid crystal induced morphological control of solid composite polymer electrolyte for lithium-ion batteries. *Mater. Des.* **2020,** 192, 108760.
42. Guan, X.; Wu, Q.; Zhang, X.; Guo, X.; Li, C.; Xu, J. In-situ crosslinked single ion gel polymer electrolyte with superior performances for lithium metal batteries. *Chem. Eng. J.* **2020**, 382, 122935.
43. He, H., Tang, C., Peng, Z., Fan, M., Jiang, M., Li, D., Kang, W., Wang, X., Deng, N., Gao, L., Zhuang, X. Enhanced mechanical strength and improved Li^+ transport in PEO-based electrolytes via scalable bicontinuous PMIA porous membrane. *J. Energy Chem.* **2026**, 112, 183–197.
44. Li, B., Liu, K., Wang, Z., Zhao, Y., Lv, Y., Shi, L., Yuan, S. An ultrathin polymer-based solid-state electrolyte membrane with high conductivity for lithium metal batteries operable at −25 °C. *J. Colloid Interface Sci.* **2025**, 698, 138071.
45. Wang, C., Duan, X., Chen, X., Weng, H., Li, M., Sun, L., Li, Y. Boosting the rate performance of all-solid-state batteries with a novel double layer solid electrolyte. *J. Energy Storage* **2024**, 101, 113730.
